# De-humanising general practice leadership: shining a light on contemporary challenges

**DOI:** 10.3399/BJGP.2026.0154

**Published:** 2026-06-01

**Authors:** Sophie Park, Ruth Abrams, Geoff Wong, Kamal R Mahtani

**Affiliations:** 1 Nuffield Department of Primary Care Health Sciences, University of Oxford, Oxford, UK; 2 School of Health Sciences, University of Surrey, Surrey, UK

General practice is deeply personal work. It relies on building relationships with patients, colleagues, and communities, and on the skilled navigation of complexity, uncertainty, and trust.^1^ Yet much of the current policy discourse focuses on workforce attrition and access metrics.^2,3^ In response to sustained pressure over the past decade including recent GP contract changes, many practices have adopted increasingly industrial strategies to survive: mergers into larger organisations, sale to commercial management companies, and rapid standardisation alongside technological expansion.^4^

These shifts are often framed as economic necessities. Human concerns — patient care and advocacy, workforce wellbeing, professional autonomy — are rhetorically positioned in opposition to financial sustainability. The evidence suggests otherwise. Organisations that align economic decision making with human priorities tend to perform better over time.^5–7^ Don Berwick’s description of ‘Era 2’ cultures — dominated by measurement, standardisation, and top-down accountability — contrasts with an ‘Era 3’ mindset that emphasises learning, relationships, and shared purpose to improve care rather than to control it.^8^

*“When organisational ease is prioritised over human concern, colleagues and patients risk being discussed as problems to be managed rather than people to be understood.”*

Era 2 responses are understandable: faced with escalating demand and shrinking margins, standardisation promises uniformity, efficiency, and risk reduction. However, when large and insufficiently adapted partnership groups rely heavily on standardisation and top-down accountability, there is a danger that practice interactions — both clinical and organisational — become de-humanised. Fear of risk, regulatory scrutiny, and financial instability fosters conformity and suppresses dissent. Subtle pressures to conform emerge as unity is perceived as a prerequisite for (economic) survival.

This tension is particularly acute within modern GP partnerships. Historically, partnerships were often small, autonomous, locally responsive, and demonstrably cost-effective.^4^ The contemporary partnership can now look very different: fifteen or more partners, complex governance arrangements, and layers of managerial infrastructure. Despite this scale, decision making often remains rooted in assumptions designed for much smaller groups: collective deliberation, expectations of universal agreement, and informal mechanisms of accountability.

This creates a striking dissonance. Clinically, GPs practise expert generalism: they individualise care, hold multiple perspectives, and embrace uncertainty. They value curiosity, critical thinking, and humility in shared decision making.^1^ Yet, as partners within large organisations, the environment rewards compliance, where to challenge dominant narratives — about finance, productivity, or workforce management — feels risky. Silence may appear safer than dissent.

The consequences matter. Research on the ‘dark side’ of leadership and on intersectional inequalities in medicine reminds us that cultures of fear, informal hierarchies, and unexamined bias are neither rare nor benign.^9,10^ Where psychological safety is low, bullying and exclusion can thrive.^11^ When organisational ease is prioritised over human concern, colleagues and patients risk being discussed as problems to be managed rather than people to be understood. Emotional withdrawal becomes a coping mechanism. Objectification follows.

Such dynamics threaten the very foundations of general practice. Collective decision making and shared accountability are strengths in small, cohesive groups. In larger, more heterogeneous partnerships, we must adapt. Without explicit attention to how power operates, disagreements are handled, and diverse perspectives are integrated, even well-intentioned governance slides into depersonalisation.

## What does this mean in 2026?

General practice is not simply a business — driven by efficiency and profit — but often the patients’ first place to get help. Standardisation may bring short-term benefits, such as conformity and consistency of care; however, focusing solely on standardisation inevitably means you serve both your patients and your workforce less well. Instead, The King’s Fund research advises that economic and human needs are not competing demands: to be compassionate is, counterintuitively, to be more effective and efficient.^5^

Larger practices should therefore consciously embrace different opinions at practice meetings. For example, a staff member may have found a group of patients that do not need or want the practice’s standard patient journey for high blood pressure management. Instead of dismissing that opinion (and, potentially, that group of patients) as non-conforming, a practice needs to recognise dissent as a useful potential route to better care. This requires practices to trust and distribute authority across staff networks, allowing staff discretion to meet (often unpredicted or new) patient needs. Distributed leadership requires greater exchange of power, agency, and responsibility to achieve agreed outcomes. Indeed, practices where staff can be innovative and creative are more likely to adapt to challenges effectively and at scale.^12,13^

The ability of a collective network to function at scale requires sustained investment and attention. This includes regular discussions on how the practice values are operationalised. For example, if the practice values Marmot’s ‘proportionate universalism’*,* ^14^ it might give agency to staff to make specific adaptations for individual patients. It’s a short-term cost, but likely to be more cost-effective to the NHS as a whole, as well as making care more equitable.

*“... practices where staff can be innovative and creative are more likely to adapt to challenges effectively and at scale.”*

General practice stands in a liminal space: applying a model developed for small-scale practices with minimal modification to increasingly large and complex institutions. Other sectors (such as pharmacy) have navigated similar transitions, but context matters. The relational core of general practice for patients and staff — continuity, trust, local accountability — is not incidental but central to its effectiveness.

If partnerships are to survive and flourish, they must evolve not only structurally but culturally. Mistaken as luxuries, intentional cultivation of reflective, inclusive leadership; protected spaces for critical dialogue; and alignment of economic decisions with human values are prerequisites for a humane and sustainable future.
